# The effect of counseling based on health promotion awareness on self-care needs and reproductive and sexual health literacy of newly married women: a randomized controlled clinical trial study

**DOI:** 10.1186/s12905-024-03214-9

**Published:** 2024-06-28

**Authors:** Fereshteh Kohansal, Roghaiyeh Nourizadeh, Niloufar Sattarzadeh Jahdi, Mahdie Arab Bafrani, Esmat Mehrabi

**Affiliations:** 1 Department of Midwifery, Faculty of Nursing and Midwifery, Tabriz University of Medical Sciences, Tabriz, Iran; 2 School of Nursing and Midwifery, Isfahan University of Medical Sciences, Isfahan, Iran

**Keywords:** Health promotion, Self care, Health literacy, Reproductive health, Married women

## Abstract

**Background:**

Despite the importance of health literacy and the self-care skills in improving individual and social health and health costs reduction, scientific evidence indicates women’s poor awareness of self-care needs and low health literacy concerning reproductive and sexual health in most societies. The present study was conducted to specify the effect of health awareness promotion on self-care needs and reproductive and sexual health literacy of newly married women.

**Methods:**

This randomized controlled clinical trial was conducted on 64 newly married women aged 15–45 in Tehran, Iran from August 2021 to the end of December 2021. The participants were randomly assigned into the intervention (*n* = 32) and control (*n* = 32) groups. The intervention group received four individual health awareness-promotion education sessions. The reproductive and sexual self-care needs, and sexual health literacy questionnaires, were completed before and 4-week after the intervention through interview. The data were analyzed using SPSS26 software. The independent t-tests and ANCOVA were used to comparison the mean scores and a significance level of *P* < 0.05 was considered.

**Results:**

The results of this study indicated that after counseling, the average overall score of perceived reproductive and sexual self-care needs significantly decreased in the intervention group [Mean (standard deviation(SD)): 125.70 (24.70)] compared to the control group [Mean (SD): 87.1 (23.42)][*P* = 0.001]. Also, the mean score of sexual and reproductive health literacy significantly increased in the intervention group [Mean (SD): 125.50 (14.09)] compared to the control group [Mean (SD): 97.15 (14.90)] after intervention [*P* = 0.01].

**Conclusions:**

The results indicated the positive effect of health promotion awareness educations on reproductive and sexual self-care needs and health literacy among newly married women. Therefore, health promotion interventions should be incorporated in health services provision programs for newly married women in comprehensive health centers to improve the health of women and families.

**Trial registration:**

Iranian Registry of Clinical Trials (IRCT): IRCT20171007036615N7 Date of registration: 2021-09-21. URL: https://fa.irct.ir/trial/58597.

## Background

Sexual and reproductive health is considered as one of the main and very important aspects of individual health [1], as the rapid and universal access to reproductive health services, information, and education has been emphasized in the international conference on population and development to improve reproductive and sexual health [2, 3]. It is estimated that about 4.3 million sexually active persons worldwide will receive poor and/or limited access to Sexual and Reproductive Health (SRH) services in their lifetime and unfortunately, myriad Sexual and Reproductive Health and Rights (SRHR) agenda gaps remain unaddressed [4]. In general, reproductive health is defined as the state of complete physical, mental, and social well-being in relation to the reproductive system and its function [5].

Sexual health education programs always emphasize on improving the sexual health literacy level and reducing sexual problems among different groups of society [6]. Sexual health literacy is defined as a set of personal knowledge, attitudes, beliefs, motivations, and abilities in accessing, understanding, evaluating, and using sexual health-related information [7]. Enjoying a desirable level of sexual health literacy increases the ability to analyze, judge, discuss, decide, and change sexual behavior [8–10] and and improve sexual interactions, individual sexual health, and family and social health [9, 10].

Self-care performance refers to the conscious, learned, voluntary and purposeful actions and activities based on perceived needs, which individuals do to preserve life, improve the health of themselves and their family, prevent the occurrence of illness, and restore health after illness and injury [11]. In this process, the necessary measures are taken by the individual herself, and health care providers are only responsible for providing information and guidance to lessen the perceived self-care needs and improve the self-care performance among individuals [12, 13]. Some benefits of self-care are the reduction of the re-hospitalization and health costs, enhancement of the adaptation to the disease, improvement of the quality of life [14], empowerment, self-confidence, and informed decision-making [13]. Countries can use interventions to promote self-care as gateways for more people to access quality, accessible, and equitable services, which are essential for achieving universal health coverage (UHC) [13].

Limited studies have examined the level of sexual health literacy and self-care, and the level of sexual health literacy among Iranian women has been reported to be unfavorable and at the borderline level (56.4%) [15, 16].

Iranian women face unique challenges when it comes to sexual health, including cultural norms, limited comprehensive sex education, and the sense of stigma and discrimination from their families, communities, or healthcare providers when they seek information or services related to sexual health. This can prevent them from seeking the care they need [17–19]. But in recent years, there has been a growing awareness of the importance of sexual health among Iranian women. This has led to initiatives aimed at providing accurate information and education on topics such as contraception, STIs, and reproductive rights. The Iranian government has taken steps to improve access to reproductive health services for women, including implementing programs to increase contraceptive use and reduce maternal mortality rates. Women’s rights activists in Iran have been working to empower women to take control of their sexual health and advocate for their rights. This includes campaigns to raise awareness about issues such as domestic violence and reproductive rights [20, 21].

While Iranian women still face significant challenges when it comes to sexual health, there have been positive developments in recent years. Increased awareness, policy changes, and empowerment initiatives are helping to improve the sexual health situation of Iranian women. However, more attempt is needed to ensure that all women have access to comprehensive sexual health services, information and appropriate and effective counseling. Providing training in the field of reproductive and sexual health is the first and most basic step to increase the necessary knowledge and skills to make informed decisions and benefit from self-care measures [22]. Evidently, different health related interventions variously influence health issues and determining the most effective and appropriate method can play a significant role in reducing costs as well as designing and implementing health education interventions [23]. Health awareness promotion, as one of the educational approaches, seems to be helpful in this field. The World Health Organization (WHO) (1986) in the Ottawa Charter formally defined awareness-raising as “the process of empowering people to control and improve their own health”. In this charter, enablement, mediation, and advocacy are mentioned as three essential health promotion strategies to achieve action areas [24]. Several studies demonstrated the effect of awareness promotion based interventions on various aspects of women’s health, such as breast cancer screening and its early detection [25–27], cervical cancer screening [28], prenatal care [29], and mother and baby care [30]. However, studies were not found that directly investigated counseling intervention approaches to improve reproductive and sexual health and the effect of health awareness-raising interventions on women’s sexual self-care needs, requires further investigations.

Since empowering women of reproductive age for taking care of themselves to provide and guarantee their health and that of their families is one of the challenges of maternal and child health care providers, this category of needs of women of reproductive age should be met using appropriate intervention approaches to improve the health and quality of life of women and their families. Considering the lack of studies in this field, the present study aimed at determining the health promotion awareness on self-care needs and sexual health literacy of newly married women.

## Methods

### Study type and participants

This randomized controlled clinical trial study was conducted on 64 married women aged 15–45 referred to comprehensive health centers in Tehran, Iran from August 2021 to the end of December 2021. The newly married women aged (at least six months passed since their marriage) with sexual and reproductive self-care needs score of more than 142.5 and reading and writing literacy were included in the study. The exclusion criteria were cancer disease or its history, history of participating in previous educational and interventional programs related to sexual and reproductive health, mental disability and any mental illness according to the individual’s health profile.

The reproductive and sexual health literacy and self-care needs were primary outcomes of present study.

### Sample size

The sample size was calculated using the mean difference formula with G.POWER software. Based on the study results of a pilot study on 20 subjects and considering M1 = 175.98 (mean score of reproductive and sexual health literacy), M2 = 140.98 (assuming a 20% reduction due to the intervention), SD1 = SD2 = 35.67, Two-sided α = 0.05, and Power = 80%, the sample size was obtained 28 per group. Also, given M1 = 80.81 (mean score of reproductive and sexual self-care), M2 = 96.97 (assuming 20% increase due to intervention), SD1 = SD2 = 14.16, Two- sided α = 0.05 and Power = 80%, the sample size of 21 was estimated. Owing to the high sample size based on reproductive and sexual health literacy variable concerning 10% attrition, the sample size was considered 32 for each group.

### Sampling and random assignment

Sampling was done after obtaining permission from the Ethics Committee of Tabriz University of Medical Sciences (IR.TBZMED.REC.1400.020) and comprehensive health centers of Tehran province from August 2021 to the end of December 2021. Tehran has 185 comprehensive health centers and the information of all women, including contact number and address, is available in these centers. After selecting 20 centers of crowded health care centers from different restrictions of Tehran, the researcher attended the selected comprehensive health centers and extracted a list of all newly married young women aged 15–45 (starting their marital life in the last six months) along with their phone numbers and addresses, convenience sampling was done from each center, and then people who met the inclusion criteria and were willing to participate in the study were randomly assigned to two intervention and control groups. Then, the eligible women were called and briefly explained the objectives and method of the research, and they were asked to attend the health center at a certain time if they intended to participate in the study. In the first introductory session, after providing a complete explanation about the method and objectives of the research and assessing the inclusion criteria, the eligible women signed the written informed consent form to participate in the study. Afterward, women whose sexual and reproductive self-care needs score was higher than the mean score was included in the study and completed the socio-demographic and obstetric characteristics form. Participants were randomly assigned into the control and intervention groups with a ratio of 1:1. For allocation concealment, the type of allocation was written on a piece of paper and put in consecutively numbered opaque envelopes. The envelopes were opened by a non-involved person in the sampling process (health center expert). The pretest questionnaires, including socio-demographic and obstetric characteristic questionnaire, reproductive and sexual self-care need assessment, and sexual health literacy were completed by the participants before the intervention.

### Intervention

The intervention group received health promotion based consultation for 40–60 min at their comprehensive health centers. Four sessions [31] were done by the first author who has MSc degree in midwifery and participated in related consultation and training courses. The content of education was prepared based on WHO Guidelines [32–34] and revised before the intervention based on 10 persons who are experts in sexual and reproductive health and the necessary amendments were made based on their opinions. All sessions were done face-to-face and individual in accordance with the quarantine protocol (the use of masks, alcohol, gloves, and social distancing) during the covid-19 pandemic. The content was presented to the intervention group in a scheduled manner during four consecutive weeks, and one to two more sessions were held if each person needed it. Similarly, the control group received four educational sessions on prenatal mental health (according to the conditions of the Covid-19 pandemic and providing relevant educational materials) for an average of 60–90 min.

In the present study, the content of consultation sessions was as follows (Table 1).

**Table 1:** The content of consultation sessions

Title of session	Purpose	Contents
**First session** self-care needs & knowledge	-Identification of participants’ self-care needs -Examining the level of reproductive and sexual awareness -Expressing the importance of promoting self-care and health literacy	• Introduction and explanation of the objectives of the research • Listening to the women’s needs in relation to reproductive and sexual issues • Communication and discussion about the types of perceived sexual self-care needs • Providing education in line with reproductive health • Identifying the sources of obtaining information in the participants and introducing reliable sources • Ask participants to record their experiences of self-care for the next session.
**Second session** personal health & self-care	-Expressing and training the use of self-care in personal health issues	• Reviewing the assignments of the previous session and discussing them • Consultation about the necessary cares for sexually transmitted diseases, menstrual cycle, urinary tract infection (prevention and treatment) • Consultation about the method of prevention and screening for breast and cervical cancer and ovarian cysts • Requesting to record the performed screenings and self-care measures in the relevant issues by the participants for the next session.
**Third session** reproductive health & health literacy	-promoting awareness on reproductive and sexual health literacy	• Reviewing the assignments of the previous session and discussing them • Consultation about various aspects of reproductive and sexual issues (reporting sexual violence, controlling unplanned pregnancy, nutrition and physical activity in maintaining reproductive health, identifying high-risk behaviors) and prevention methods • Requesting to record the self-care-related material in the relevant topics by the participants for the next session
**Fourth session** marital relationship & health literacy	-Expressing the importance of life skills in maintaining marital relationship	• Reviewing the assignments of the previous session and discussing them • Consultation about the marital relationship and life skills after marriage (effective communication, empathy and emotional intelligence, conflict resolution, patience and forgiveness, mutual respect, responsibility and sexual relationship) • Summarizing the topics presented in the sessions and checking the achievement of the research objectives.

### Data collection tools

The questionnaires of socio-demographic and obstetric characteristics, reproductive and sexual self-care need assessment, and sexual health literacy were used to collect data before and 4-week after the intervention.

The socio-demographic and obstetric characteristics form included the items of age, number of pregnancies, number of births, level of education, employment status, household income adequacy, etc.

### Sexual and reproductive self-care educational needs questionnaire

The reproductive and sexual self-care needs were assessed using a 57-item instrument designed and psychometrically evaluated by Hashemi et al. (2020) in Iran. This questionnaire includes 13 sub-domains scored based on 5-point Likert scale ranging from very high [5] to very low [1]. A high score indicates higher educational need [35]. The reliability of the questionnaire in Iran has been confirmed in present study. The Cronbach’s alpha coefficient has been calculated to be 0.91.

### Reproductive and sexual health literacy questionnaire

Sexual and reproductive health literacy was investigated using a 40-item instrument developed and psychometrically evaluated by Masoumi et al. (2017) in Iran. This questionnaire is developed for applying among Iranians and according to Iranian culture. This instrument consists of four subdomains, including access skills (7 items), reading and understanding (18 items), evaluation and analysis (5 items), and information application (10 items). The items are scored on 5-point Likert scale ranging from completely agree [5] to completely disagree (0). Its internal consistency has been confirmed for all areas with an alpha of 95% and appropriate reliability among the Iranians [36].

### Data analysis

**Fig. 1:**
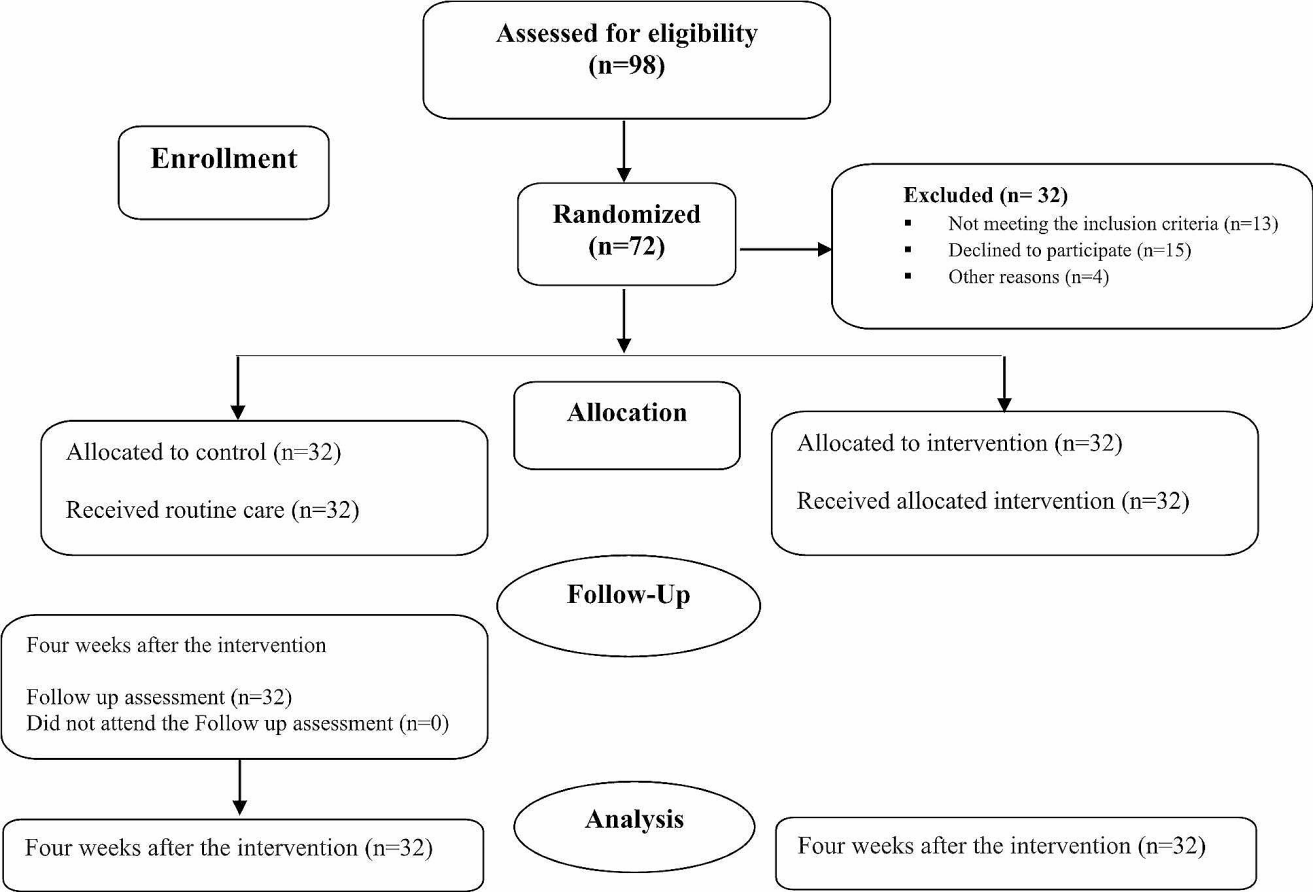
Flowchart of the study

Figure 1 shows the flowchart of the study. After evaluating the inclusion criteria and willingness to participate in the study, among a total of 96 newly married women aged 15–45, 64 women were included in the study and were randomly assigned into the intervention (*n* = 32) and control (*n* = 32) groups. Attrition did not occur during the study and 4 weeks of follow-up after the end of the sessions. The data were analyzed using SPSS26 software. The normality of the distributions was determined using the Kolmogorov-Smirnov test, and the frequency, central indices and dispersion, chi-square and independent t-tests were used to examine and compare the socio-demographic and obstetric characteristics. Independent t-test before the intervention and ANCOVA after the intervention were used to compare the mean score of the study outcomes. A significance level of *P* < 0.05 was considered.

## Results

There was no significant difference in the socio-demographic and obstetric characteristics between the control and intervention groups (*p* < 0.05). The mean age of the participants in the intervention and control groups was 25.40 (3.27) and 24.68 (3.42) years, respectively. The educational degree of more than half of the women in the studied groups was diploma. Almost all women participating in the intervention and control groups were housewives (Table 2).

Before the intervention, the results of the independent t-test showed no significant difference in the total score of reproductive and sexual self-care needs and its components between the intervention and control groups and the mean difference between two studied group was not significant (*P* = 0.39). The mean score of reproductive and sexual self-care educational needs was statistically and significantly reduced in the intervention group compared to the control group 4 weeks after the intervention [MD: -38.6, 95% CI: -20.10 to -10/57, *P* = 0.001]. also the mean score of self-care needs was significantly lower than control group after intervention (Table 3). Further, the analysis of data related to health literacy showed that the total mean score of reproductive and sexual health literacy in the intervention group compared to the control group increased significantly 4 weeks after the intervention. [MD: 27.35, 95% CI: 15.00 to 39.70, *P* < 0.01].(Table 4).

**Table 2:** The socio-demographic and obstetric characteristics of the participants

Variables		Intervention group Number (%) (*n* = 32)	Control group Number (%) (*n* = 32)	*P*-value
Age (year)		25.40 (3.27)^*^	24.68 (3.42)^*^	0.26^**^
Spose’s age		28.00 (4.29)^*^	28.43 (4.67)^*^	0.15^**^
Educational level	< Diploma	7 (21.8)	6 (18.7)	0.26^¥^
	Diploma	20 (62.5)	22 (67.7)	
	Bachelor degree and higher	5 (15.6)	4 (12.5)	
Spouse’s education	< Diploma	7 (21.8)	6 (18.7)	0.34^¥^
	Diploma	16 (50.0)	18 (56.2)	
	Bachelor’s degree and higher	9 (28.1)	8 (25.0)	
Employment status	Housewife	30 (93.8)	29 (90.7)	1.00^¥^
	Employed	2 (6.2)	3 (9.3)	
Spouse’s employment status	Worker	5 (15.6)	5 (15.6)	0.12^¥^
	Employee	7 (21.8)	6 (18.7)	
	Others (peddlers, shopkeepers, self-employed)	20 (62. 5)	21 (65.6)	
Household income level	Sufficient	2 (6.2)	3 (9.3)	0.44^¥^
	Somewhat sufficient	25 (78.1)	23 (71.8)	
	Insufficient	5 (15.6)	6 (18.7)	
Insurance status	Yes	30 (93.8)	28 (90.6)	0.25^¥^
	No	2 (6.2)	4 (9.4)	
Sources of obtained information on reproductive and sexual health	Health care personnel	8 (24.0)	7 (21.1)	0.15^¥^
	Media (radio and TV)	3 (9.3)	5 (15.6)	
	Internet and virtual networks	10 (32.6)	10 (32.0)	
	Family	4 (9.4)	2 (6.0)	
	Friends	6 (18.7)	5 (15.6)	
	Books	2 (6.0)	3 (9.7)	

_Mean (SD)*, independent t test**, Chi−Square_^¥^

**Table 3:** Comparing the mean (SD) of educational needs of reproductive and sexual self-care in both groups before and after the intervention

Variables	Intervention Mean (SD)	Control Mean (SD)	AMD^#^ 95% CI	*P*
Educational needs of public health (5-25)				
Before intervention	16.20 (2.33)	16.93 (3.67)	-0.73 (-1.16 to 0.43)	0.76^*^
After intervention	11.39 (4.61)	16.07 (3.11)	-5.32 (-8.83 to -2.01)	0.002^**^
Educational needs of the menstrual cycle (6-30)				
Before intervention	17.05 (5.17)	17.13 (4.19)	-0.44 (-1.32 to 0.42)	0.81^*^
After intervention	11.98 (3.09)	19.80 (4.99)	-7.82 (-11.38 to -4.26)	0.001^**^
Educational needs of sexually transmitted diseases (4-20)				
Before intervention	10.44 (3.76)	11.13 (3.39)	-1.17 (-2.78 to 0.54)	0.52^*^
After intervention	10.09 (2.20)	14.09 (4.36)	-4.01 (-6.25 to -1.77)	0.81^**^
Educational needs of AIDS and hepatitis (4-20)				
Before intervention	11.89 (1.01)	10.93 (2.21)	0.87 (2.34 to -0.60)	0.91^*^
After intervention	6.12 (1.70)	11.89 (1.78)	-5.75 (-9.25 to -2.25)	0.01^**^
Educational needs of vaginal infections (4-20)				
Before intervention	12.83 (4.51)	11.79 (3.63)	1.03 (2.53 to -0.41)	0.58^*^
After intervention	8.18 (2.63)	13.15 (5.16)	-4.97 (-7.81 to -1.98)	0.001^**^
Educational needs of urinary tract infections (4-20)				
Before intervention	13.80 (1.63)	13.70 (3.13)	0.10 (0.77 to -0.57)	0.43^*^
After intervention	9.28 (4.50)	14.25 (3.57)	-4.97 (-7.14 to -2.57)	0.04^**^
Educational needs of breast cancer (4-20)				
Before intervention	16.93 (3.04)	16.03 (3.67)	0.73(1.16 to -0.43)	0.67^*^
After intervention	10.27 (3.91)	17.39 (5.11)	-7.23 (-10.63 to -4.01)	0.001^**^
Educational needs of cervical cancer (4-20)				
Before intervention	12.79 (3.41)	12.59 (2.42)	0.20 (1.84 to -1.44)	0.70^*^
After intervention	7.34 (5.63)	12.08 (4.00)	-4.77 (-7.47 to -1.93)	0.001^**^
Educational needs of ovarian cysts (4-20)				
Before intervention	12.08 (3.37)	12.29 (2.10)	0.11 (0.78 to -0.56)	0.34^*^
After intervention	6.65 (1.60)	12.38 (2.59)	-6.49 (-10.60 to -2.38)	0.001^**^
Educational needs of sexual violence (3-15)				
Before intervention	12.01 (4.79)	12.75 (4.31)	0.76 (1.20 to -0.32)	0.28^*^
After intervention	5.85 (2.51)	13.10 (5.59)	-7.25 (-11.48 to -3.02)	0.001^**^
Educational needs of sexual health (6-30)				
Before intervention	18.72 (4.79)	18.60 (4.26)	0.12 (2.16 to -0.48)	0.67^*^
After intervention	13.11 (3.70)	19.35 (3.19)	-6.24 (-10.14 to -2.44)	0.03^**^
Educational needs of pregnancy (6-30)				
Before intervention	18.31 (5.66)	18.95 (6.70)	0.65 (2.82 to -1.54)	0.44^*^
After intervention	15.27 (4.18)	18.55 (4.70)	-3.28 (-13.16 to -4.60)	0.001^**^
Educational needs of high-risk behaviors (2-10)				
Before intervention	8.25 (1.70)	8.20 (1.35)	0.05 (0.75 to -0.65)	0.18^*^
After intervention	5.35 (1.00)	8.28 (1.32)	-2.93 (4.14 to 1.72)	0.001^**^
Total score (57–285)				
Before intervention	125.80 (24.80)	124.65 (25.14)	1.56 (2.80 to -0.50)	0.39^*^
After intervention	125.70 (24.70)	87.1 (23.42)	-38.6 (-57.10 to -20.10)	0.001^**^

* t-test, ** ANCOVA test by controlling baseline effect, # adjusted mean difference with 95% confidence interval

**Table 4:** Comparing the mean (standard deviation) of reproductive and sexual health literacy and its subdomains in the intervention and control groups, before and after the intervention

Variables	Intervention Mean (SD)	Control Mean (SD)	AMD^#^ (95%CI)	*P*
Skill and access (0–35)				
Before intervention	21.18 (3.30)	21.34 (4.10)	0.84 (1.87 to -0.51)	0.45^*^
After intervention	29.24 (3.12)	21.34 (3.80)	7.90 (13.56 to 2.24)	0.02^*^
Reading and understanding(0–90)				
Before intervention	43.50 (15.27)	42.90 (14.11)	0.60 (1.41 to -0.23)	0.22^*^
After intervention	58.85 (17.77)	41.51 (14.56)	17.34 (26.26 to 8.42)	0.001^**^
Evaluation and analysis (0–25)				
Before intervention	13.40 (4.23)	12.90 (4.45)	0.50 (2.24 to -0.23)	0.51^*^
After intervention	18.85 (5.89)	13.28 (5.33)	5.57 (8.27 to 2.87)	0. 01^**^
Information application(0–50)				
Before intervention	23.80 (4.23)	24.80 (3.09)	-0.33 (-1.30 to 0.64)	0. 14^*^
After intervention	29.54 (3.59)	23.95 (2.09)	5.58 (8.34 to 2.84)	0.04^**^
Total score of reproductive and sexual health literacy (0-200)				
Before intervention	98.37 (9.23)	97.20 (11.95)	1.17 (2.68 to -0.34)	0.68^*^
After intervention	124.50 (14.09)	97.15 (14.90)	27.35 (39.70 to 15.00)	0.01^**^

* t-test, ** ANCOVA test by controlling baseline effect, # adjusted mean difference with 95% confidence interval

## Discussion

Based on the findings, providing health awareness promotion counselling can reduce the reproductive and sexual self-care educational needs of newly married women in all areas and increase the reproductive and sexual health literacy level. Promoting sexual and reproductive health, especially in women of reproductive age, is very important and it can improve the global health indicators, such as maternal mortality.

In the present study, the mean score of self-care educational needs in the intervention group after receiving consultation, decreased significantly. There was not RCT studies in relation to present study outcomes, and the cross-sectional and also qualitative studies applied for discussion. In consistent with this result, Phulambrikar et al., in a systematic review reported that educational interventions can increase adolescent’s knowledge related to sexual and reproductive health [37]. Vongxay et al. also reported that the health literacy of adolescents was low [38]. These results are in consistent our results. In our study the mean score of sexual and reproductive health literacy was low before the intervention and also these findings demonstrate the necessity need for interventions which aimed to increase health literacy and self-care ability. In this regard Havaei et al. reprted that education based on protection motivation theory can effective on adolescents’ reproductive health self-care [39]. These result are in consistency with present study but they applied kind of health education and they consider not the needs of participants in content of education. In present study participants were selected who had high score of the self-care educational needs and received special consultation.

In present study the mean score of breast and cervical cancer educational needs greatly decreased among other areas, indicating the effectiveness of the mentioned approach in meeting the educational needs in this field among the participants of the intervention group. In line with the findings of the present study, in tow randomized controlled clinical trial studies investigated the effect of interventions based on the health promotion model on the breast and cervical cancer early detection behaviors and reported that the awareness level about breast and cervical cancer increased after the intervention [26, 28].

In our study, additionally we paid attention to the domain of Sexually transmitted diseases, such as Human Papilloma Virus (HPV) and HIV which are considered as common problems in today’s society, especially among women of reproductive age. In this regard, Henderson et al. reported that behavioral based interventions are effective on the incidence of sexually transmitted infections (STIs) among adults and adolescents at risk [40]. The appropriate awareness-raising interventions in this field can have a significant impact on the financial burden of the adverse consequences of this category of diseases and consequence complications, such as cervical cancer. Therefore, the importance of paying attention and identifying the effective intervention approach to improve awareness and self-care skills in this field should be included in the health policies of the health system of the countries.

The findings of the present study revealed an improvement in the mean score of self-care educational needs during menstruation and pregnancy in the group receiving educational sessions. In the same vein, another studies investigated the effect of an educational intervention on the menstrual health of students and demonstrated significant changes in the knowledge and practices of menstrual health after the intervention [41]. Also, improving women’s awareness and providing health education before pregnancy can effectively prevent health problems during pregnancy [42]. Since marriage and the beginning of marital life are considered as a prelude to childbearing, it is necessary to apply the results of the present study and other similar studies in this field in the form of training packages through face-to-face or virtual training, presentation of brochures, health messages, etc.

In the present study, the score of sexual and reproductive health literacy in four areas of skill and access, reading and understanding, evaluation and analysis, and application of information increased significantly after providing educations to the intervention group. Consistent with the results of the present study, Rastegar et al. [43] reported that the participants’ self-care performance improved by increasing the mean score of health literacy. In addition, Zhuang et al. indicated that a cell phone-based health education intervention improved health literacy [44]. Considering that the high level of health literacy among women in the society can improve the quality of prenatal care, increase planned pregnancies, and decrease treatment costs, it is recommended to pay more attention to educational interventions for reproductive age women to increase their health literacy.

### Strengths and limitations

One of the strengths of this study is the observance of all principles of randomized controlled trials, including random allocation and allocation concealment. Also, the psychometric characteristics of applied questionnaires have already been investigated in Iran. The generalizability of the results with more confidence, due to the selection of participants from all comprehensive health centers in Tehran, the completion of questionnaires in the form of interview, and the reduction of possible problems are also considered as the strengths of the present study. One of the limitations of this study was that it coincided with the COVID-19 pandemic and some participants refused to participate in the study, which was tried to comply with the hygiene protocol and use gloves and masks and social distance in providing training and education to some extent.

## Conclusion

In general, the results indicated the effect of health awareness promotion consultation on improving awareness, health literacy level, and the reproductive and sexual self-care skills of women. Present study supplied the data in relation to reproductive health for reproductive aged women who the large number of people in the world. Empowering women in the field of reproductive and sexual self-care reduces unnecessary referrals to the specialized department and financial costs for the individual and the country. Because woman, as an important member of the family, can play an important role in improving the health and primary care of other family members by learning the principles of self-care. As a practical recommendation, the health policy makers should provide an interventional package for Promoting women’s self-care especially regarding the reproductive and sexual health-related issues so that they are not misguided by any unreliable sources of information. Age and sex appropriate health consultations can facilitate the development of healthy reproductive and sexual behavior patterns among women through enhancement of knowledge and development of right attitude.

## Data Availability

Data sets used and/or analyzed during the current study are available from the corresponding author upon reasonable request.
